# Optimisation of engineered *Escherichia coli* biofilms for enzymatic biosynthesis of l-halotryptophans

**DOI:** 10.1186/2191-0855-3-66

**Published:** 2013-11-04

**Authors:** Stefano Perni, Louise Hackett, Rebecca JM Goss, Mark J Simmons, Tim W Overton

**Affiliations:** 1 School of Chemical Engineering, University of Birmingham, Birmingham B15 2TT, UK; 2 School of Chemistry, University of St. Andrews, St Andrews, Fife KY16 9ST, UK

**Keywords:** *E. coli*, Biofilm, Biotransformation, Haloindole, Halotryptophan

## Abstract

Engineered biofilms comprising a single recombinant species have demonstrated remarkable activity as novel biocatalysts for a range of applications. In this work, we focused on the biotransformation of 5-haloindole into 5-halotryptophan, a pharmaceutical intermediate, using *Escherichia coli* expressing a recombinant tryptophan synthase enzyme encoded by plasmid pSTB7. To optimise the reaction we compared two *E. coli* K-12 strains (MC4100 and MG1655) and their *ompR234* mutants, which overproduce the adhesin curli (PHL644 and PHL628). The *ompR234* mutation increased the quantity of biofilm in both MG1655 and MC4100 backgrounds. In all cases, no conversion of 5-haloindoles was observed using cells without the pSTB7 plasmid. Engineered biofilms of strains PHL628 pSTB7 and PHL644 pSTB7 generated more 5-halotryptophan than their corresponding planktonic cells. Flow cytometry revealed that the vast majority of cells were alive after 24 hour biotransformation reactions, both in planktonic and biofilm forms, suggesting that cell viability was not a major factor in the greater performance of biofilm reactions. Monitoring 5-haloindole depletion, 5-halotryptophan synthesis and the percentage conversion of the biotransformation reaction suggested that there were inherent differences between strains MG1655 and MC4100, and between planktonic and biofilm cells, in terms of tryptophan and indole metabolism and transport. The study has reinforced the need to thoroughly investigate bacterial physiology and make informed strain selections when developing biotransformation reactions.

## Introduction

Bacterial biofilms are renowned for their enhanced resistance to environmental and chemical stresses such as antibiotics, metal ions and organic solvents when compared to planktonic bacteria. This property of biofilms is a cause of clinical concern, especially with implantable medical devices (such as catheters), since biofilm-mediated infections are frequently harder to treat than those caused by planktonic bacteria (Smith and Hunter, 2008). However, the increased robustness of biofilms can be exploited in bioprocesses where cells are exposed to harsh reaction conditions (Winn *et al.,*^2012^). Biofilms, generally multispecies, have been used for waste water treatment (biofilters) (Purswani *et al.,*^2011^; Iwamoto and Nasu, 2001; Cortes-Lorenzo *et al.,*^2012^), air filters (Rene *et al.,*^2009^) and in soil bioremediation (Zhang *et al.,*^1995^; Singh and Cameotra, 2004). Most recently, single species biofilms have found applications in microbial fuel cells (Yuan *et al.,*2011a; Yuan *et al.,*2011b) and for specific biocatalytic reactions (Tsoligkas *et al.,*^2011^; Gross *et al.,*^2010^; Kunduru and Pometto, 1996). Recent examples of biotransformations catalysed by single-species biofilms include the conversion of benzaldehyde to benzyl alcohol (*Zymomonas mobilis*; Li *et al.,*^2006^), ethanol production (*Z. mobilis* and *Saccharomyces cerevisiae*; Kunduru and Pometto, 1996), production of (*S*)-styrene oxide (*Pseudomonas* sp.; Halan *et al.,*^2011^; Halan *et al.,*^2010^) and dihydroxyacetone production (*Gluconobacter oxydans*; Hekmat *et al.,*^2007^; Hu *et al.,*^2011^).

When compared to biotransformation reactions catalysed by purified enzymes, whole cell biocatalysis permits protection of the enzyme within the cell and also production of new enzyme molecules. Furthermore, it does not require the extraction, purification and immobilisation involved in the use of enzymes, often making it a more cost-effective approach, particularly upon scale-up (Winn *et al.,*^2012^). Biofilm-mediated reactions extend these benefits by increasing protection of enzymes against harsh reaction conditions (such as extremes of pH or organic solvents) and offering simplified downstream processing since the bacteria are immobilised and do not require separating from reaction products. These factors often result in higher conversions when biotransformations are carried out using biofilms when compared to purified enzymes (Winn *et al.,*^2012^; Halan *et al.,*^2012^; Gross *et al.,*^2012^).

**Figure 1:**
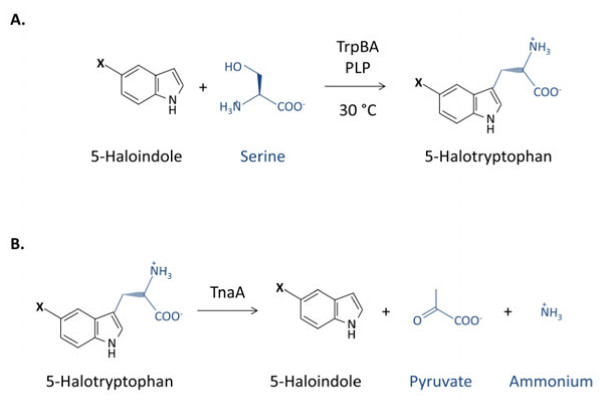
**Formation and breakdown of 5-halotryptophan in *E. coli*. (a)** Reaction scheme for biocatalytic conversion of 5-haloindole and serine to 5-halotryptophan, catalysed by tryptophan synthase TrpBA. **(b)** Reaction scheme for the reverse reaction, catalysed by tryptophanase TnaA. X = F, Cl or Br.

To generate a biofilm biocatalyst, bacteria must be deposited on a substrate, either by natural or artificial means, then allowed to mature into a biofilm. Deposition and maturation determine the structure of the biofilm and thus the mass transfer of chemical species through the biofilm extracellular matrix, therefore defining its overall performance as a biocatalyst (Tsoligkas *et al.,*^2011^; ^2012^). We have recently developed methods to generate engineered biofilms, utilising centrifugation of recombinant *E. coli* onto poly-l-lysine coated glass supports instead of waiting for natural attachment to occur (Tsoligkas *et al.,*^2011^; ^2012^). These biofilms were used to catalyse the biotransformation of 5-haloindole plus serine to 5-halotryptophan (Figure 1a), an important class of pharmaceutical intermediates; this reaction is catalysed by a recombinant tryptophan synthase TrpBA expressed constitutively from plasmid pSTB7 (Tsoligkas *et al.,*^2011^; ^2012^; Kawasaki et al. 1987). We previously demonstrated that these engineered biofilms are more efficient in converting 5-haloindole to 5-halotryptophan than either immobilised TrpBA enzyme or planktonic cells expressing recombinant TrpBA (Tsoligkas *et al.,*^2011^).

In this study, we further optimised this biotransformation system by investigating the effect of using different strains to generate engineered biofilms and perform the biotransformation of 5-haloindoles to 5-halotryptophans. Engineered biofilm generation was tested for four *E. coli* strains: wild type K-12 strains MG1655 and MC4100; and their isogenic *ompR234* mutants, which overproduce curli (adhesive protein filaments) and thus accelerate biofilm formation (Vidal et al. 1998). Biofilms were generated using each strain with and without pSTB7 to assess whether the plasmid is required for these biotransformations as *E. coli* naturally produces a tryptophan synthase. The viability of bacteria during biotransformation reactions was monitored using flow cytometry. We also studied the biotransformation reaction with regard to substrate utilisation, product synthesis and conversion efficiency to allow optimisation of conversion and yield. This constitutes an essential step forward which will provide knowledge to future practitioners wishing to scale up this reaction.

## Materials and Methods

### Strains, biofilm generation and maturation

pSTB7, a pBR322-based plasmid containing the *Salmonella enterica* serovar Typhimurium TB1533 *trpBA* genes and encoding ampicillin resistance (Kawasaki *et al.,* 1987), was purchased from the American Type Culture Collection (ATCC 37845). *E. coli* K-12 strains MG1655 (λ - F - prototroph), PHL628 (MG1655 *malA*-*kan ompR234*; Vidal et al. 1998), MC4100 (*araD139Δ(argF-lac)U169 rpsL150 relA1 flbB5301 deoC1 ptsF25 rbsR*) and PHL644 (MC4100 *malA*-*kan ompR234*; Vidal et al. 1998) were employed in this study. All *E. coli* strains were transformed with pSTB7 using the heat-shock method. Transformants were selected on Luria-Bertani-agar (10 g L^-1^ tryptone, 5 g L^-1^ yeast extract, 10 g L^-1^ NaCl, 15 g L^-1^ Bacteriological Agar; Sigma, UK) supplemented with ampicillin (100 μg mL^-1^). All *E. coli* strains were grown in 200 mL half strength Luria-Bertani (LB) broth (5 g L^-1^ tryptone, 2.5 g L^-1^ yeast extract, 5 g L^-1^ NaCl; Sigma, UK), supplemented with ampicillin (100 μg mL^-1^.) for pSTB7 transformants, in an orbital shaker at 30°C, 70 rpm with a throw of 19 mm for 24 hours. Engineered biofilms were generated using the spin-down method described by Tsoligkas *et al*^2011^) and available in Additional file 1.

### Biotransformations

Biotransformation reactions were carried out as previously described (Tsoligkas *et al.,*^2011^; full details in Additional file 1) using either planktonic cells or engineered biofilms in a potassium phosphate reaction buffer (0.1 M KH_2_PO_4_, 7 mM Serine, 0.1 mM Pyridoxal 5′-phosphate (PLP), adjusted to pH 7.0) supplemented with 5% (v/v) DMSO and either 2 mM 5-fluoroindole (270 mg L^-1^), 2 mM 5-chloroindone (303 mg L^-1^), or 2 mM 5-bromoindole (392 mg L^-1^). 5-chloroindole and 5-bromoindole are less soluble than 5-fluoroindole, so lower concentrations were present in the reaction buffer; around 0.7 mM for 5-chloroindole and 0.4 mM for 5-bromoindole (Additional file 1: Table S1). In each case, reaction buffer was made with an initial quantity of haloindole equivalent to 2 mM and decanted into biotransformation vessels, preventing any undissolved haloindole from entering the biotransformation. No attempt has been made to carry out the reactions at the same starting concentrations since an in-depth kinetic analysis was not the focus of this study. All biotransformations, irrespectively of the cells’ physiological state, were conducted on two or three independent cultures. Since 5-fluoroindole biotransformations were the most active, biotransformations were performed with all strain combinations. Biotransformations with 5-chloroindole and 5-bromoindole were performed with selected strains to generate indicative data.

### HPLC analysis

Haloindole and halotryptophan concentrations were measured in biotransformation samples by HPLC using a Shimadzu HPLC with a ZORBAX (SB-C18 4.6 mm × 15 cm) column resolved with methanol versus water at a rate of 0.7 mL min^-1^; a UV detector at 280 nm was used throughout the analysis (Additional file 1: Figure S1). Both solvents were acidified with 0.1% formic acid and run using the gradient described in the supplementary data. Linear standard curves (Additional file 1: Figure S2; peak area versus concentration) were generated for 5-fluoro-, 5-chloro- and 5-bromoindole and each corresponding 5-halotryptophan using standards of known concentration (0.125 mM to 2 mM) in triplicate and used to correlate sample peak area to concentration. Biotransformation data are presented as three percentages of halotryptophan yield (Y), haloindole depletion (D) and selectivity of conversion (S) for each timepoint:

(1) $$Y=\frac{\mathrm{halotryptophan}\;\mathrm{concentration}}{\mathrm{initial}\;\mathrm{haloindole}\;\mathrm{concentration}}\times 100$$

(2) $$D=\frac{\mathrm{initial}\;\mathrm{haloindole}\;\mathrm{concentration}‒\mathrm{haloindole}\;\mathrm{concentration}}{\mathrm{initial}\;\mathrm{haloindole}\;\mathrm{concentration}}\times 100$$

(3) $$S=\frac{Y}{D}\times 100$$

### Quantification of the dry cell biomass and Crystal Violet staining

The total biofilm biomass was determined for 5 slides that had been coated with *E. coli* biofilms and matured for 7 days. The glass slides were washed twice in phosphate buffer. In a pre-weighed centrifuge tube kept at 100°C overnight, the biofilm was disrupted in sterile water using a vortex mixer for 30 minutes; the glass slide was removed and the cells centrifuged at 1851 g for 10 minutes. The supernatant was removed and the biomass dried at 100°C for at least 24 hrs. The dry biomass was determined when the mass stopped decreasing.

The quantification of dry cell biomass of planktonic cells was performed directly on 10 mL of three independent cell suspensions in pre-weighed centrifuge tubes kept at 100°C overnight. Following centrifugation (1851 g for 10 minutes) and washing in sterile water, the cells were centrifuged again (1851 g for 10 minutes) and, after removing the liquid, allowed to dry at 100°C for at least 24 hours until a constant mass was reached.

Biofilms on glass slides were also quantified using Crystal Violet staining; after washing in sterile phosphate buffer the slides were coated with 1 mL of Crystal Violet solution (0.1% (w/v) for 15 min). The slides were washed in water three times and placed in Duran bottles with 20 mL of ethanol. The crystal violet on the glass slides was allowed to dissolve for 1 hour and the optical density of the ethanol solution determined at 570 nm using a UV–vis spectrophotometer.

### Flow cytometry

Cell membrane potential and membrane integrity were analysed by flow cytometry after 2 and 24 hours in each reaction condition using staining with 5 μg mL^-1^ propidium iodide (PI, which enters cells with compromised membrane integrity) and 0.1 mg mL^-1^ Bis (1,3-dibarbituric acid) trimethine oxanol (BOX, which enters cells with depolarised membranes) as previously described by Whitehead *et al.*^2011^). Cells were analysed using an Accuri C6 flow cytometer (BD, UK) as described in the Additional file 1.

## Results

### Biofilm formation by different *E. coli* strains

**Figure 2:**
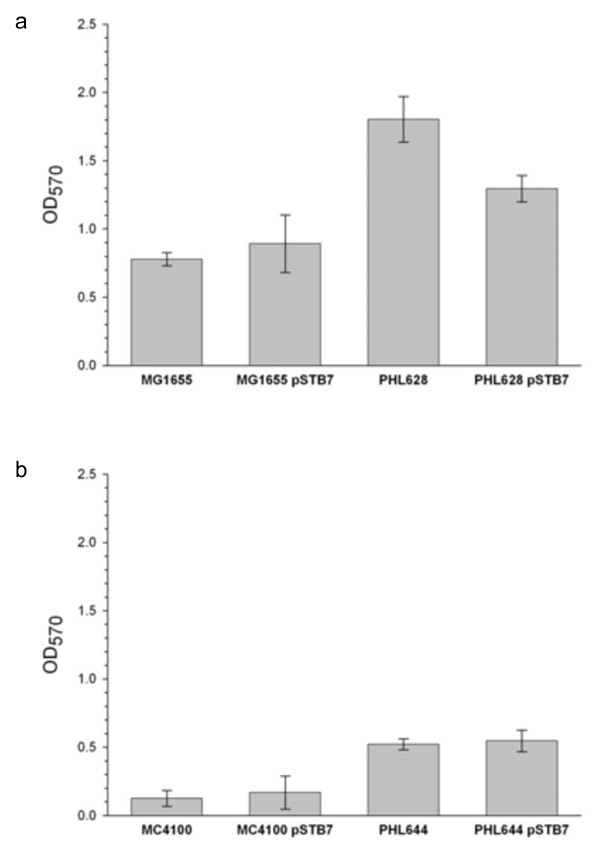
**Crystal Violet staining of *E. coli *engineered biofilms.** Biofilms were generated from strains MG1655 and PHL628 **(a)** or MC4100 and PHL644 **(b)** with and without pSTB7 using the spin-down method, matured for 7 days in M63 medium and biomass was estimated using crystal violet staining.

Crystal Violet staining was used to compare the biomass within biofilms generated using the spin-down method with four *E. coli* strains: MG1655 and MC4100; and their *ompR234* derivatives PHL628 and PHL644 (Figure 2). MG1655 generated more biofilm than MC4100, and the *ompR234* mutation increased the amount of biofilm formed by both strains. The presence of pSTB7 decreased biofilm formation by PHL628 but did not significantly affect biofilm formation by the other strains. The corresponding dry mass of each biofilm was 1.5 ± 0.2 mg for PHL644 pSTB7 and 2.3 ± 0.3 mg for PHL628 pSTB7.

### Biotransformation by planktonic cells

The ability of planktonic cells to convert 5-haloindoles to 5-halotryptophans was assessed by measuring 5-haloindole depletion, 5-halotryptophan synthesis and the selectivity of conversion of 5-haloindole to 5-halotryptophan as defined in equations 1–3. These three measurements are required since, although the conversion of haloindole plus serine to halotryptophan is catalysed by the TrpBA enzyme, halotryptophan is a potential substrate for tryptophanase (TnaA) which would convert it to haloindole, pyruvate and ammonium (Figure 1b). Alternatively, halotryptophans could be sequestered for protein synthesis (Crowley *et al*.,^2012^). Thus, selectivity of conversion to halotryptophan is a critical parameter for the reaction to be considered as a viable route for production of these compounds. Neither depletion of haloindole nor production of halotryptophan was detected when biotransformations were performed using bacteria without the pSTB7 plasmid, either planktonically or in biofilms, confirming that the constitutively expressed recombinant tryptophan synthase is required for the reaction (data not shown).

**Figure 3:**
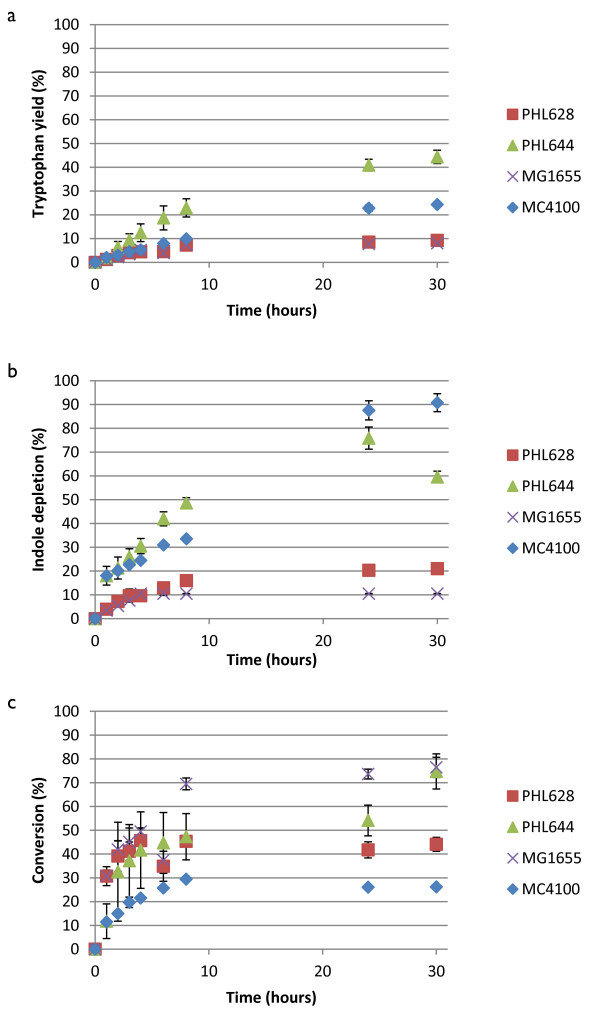
**Biotransformation of 5-fluoroindole to 5-fluorotryptophan using planktonic cells of four strains.** Concentrations of 5-fluorotryptophan and 5-fluoroindole were measured using HPLC and percentage 5-fluorotryptophan accumulation **(a)**, percentage 5-fluoroindole depletion **(b)** and the selectivity of the 5-fluoroindole to 5-fluorotryptophan reaction **(c)** were plotted against time. All cells contained pSTB7.

Figure 3a shows that the concentrations of 5-fluorotryptophan increased over the reaction period with the rate of generation decreasing as the reaction proceeded. No significant difference was noticed in synthesis rate or overall yield between MG1655 pSTB7 and PHL628 pSTB7; the rate and yield were higher for MC4100 pSTB7, and higher still for PHL644 pSTB7. The profile of 5-fluoroindole depletion (Figure 3b) appeared similar to that of 5-fluorotryptophan generation in strains MG1655 pSTB7 and PHL628 pSTB7, but displayed a rapid increase (to nearly 20%) in MC4100 pSTB7 and PHL644 pSTB7 in the first hour of the reaction. This suggests that indole efflux is much more rapid in MC4100 than in MG1655, and reflects an inherent difference between the strains. Selectivity of conversion of 5-fluoroindole to 5-fluorotryptophan increased rapidly in PHL628 pSTB7, PHL644 pSTB7 and MG1655 pSTB7, although MG1655 pSTB7 selectivity was highest after 8 hours (Figure 3c). Planktonic biotransformation reactions (in 10 mL of culture volume) contained a dry mass of 1.1 ± 0.1 mg for PHL644 pSTB7 and 1.2 ± 0.2 mg for PHL628 pSTB7.

**Figure 4:**
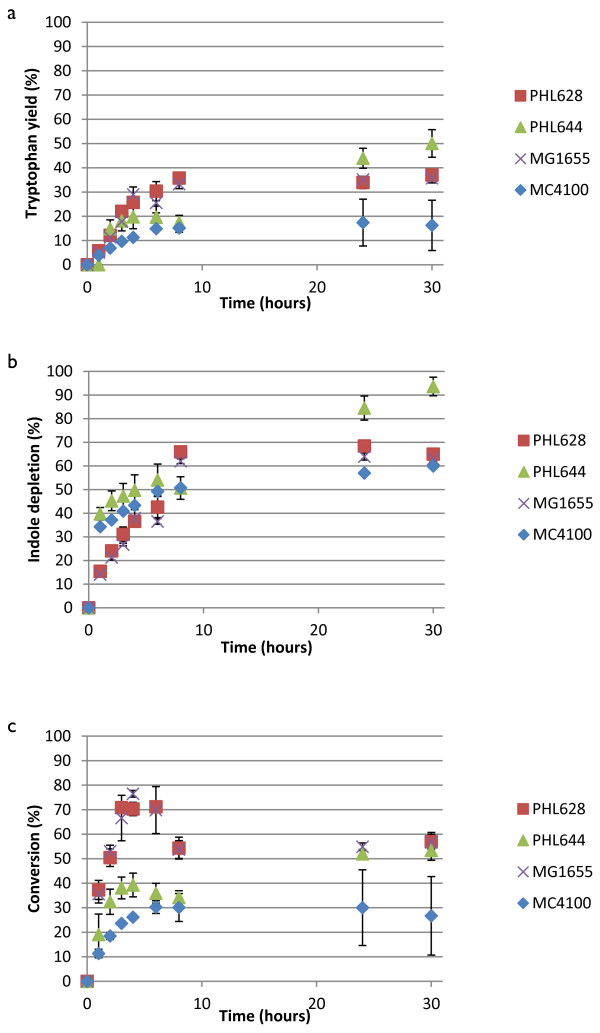
**Biotransformation of 5-chloroindole to 5-chlorotryptophan using planktonic cells of four strains.** Concentrations of 5-chlorotryptophan and 5-chloroindole were measured using HPLC and percentage 5-chlorotryptophan accumulation **(a)**, percentage 5-chloroindole depletion **(b)** and the selectivity of the 5-chloroindole to 5-chlorotryptophan reaction **(c)** were plotted against time. All cells contained pSTB7.

The same parameters are shown for the biotransformation of 5-chloroindole to 5-chlorotryptophan in Figure 4. Unlike the 5-fluoroindole reaction, strains PHL628, PHL644 and MG1655 showed similar overall percentage chlorotryptophan yields. As with the fluoroindole reactions (Figure 3), strains MC4100 pSTB7 and PHL644 pSTB7 both showed rapid chloroindole depletion in the first hour of the reaction whereas MG1655 pSTB7 and PHL628 pSTB7 displayed more gradual depletion. As a result, the selectivity of the reaction was initially higher in MG1655 pSTB7 and PHL628 pSTB7, peaking at around 75% at 4 hours, although the selectivity of these two strains decreased to around 50% over the course of the reaction. PHL644 pSTB7 selectivity increased over time to around 50% after 25 hours. As with the fluoroindole reaction, the selectivity of MC4100 pSTB7 was lowest throughout. Planktonic biotransformations yielded extremely low production of 5-bromotryptophan (>10%; Additional file 1: Figure S3). 5-bromoindole was depleted in these biotransformation reactions (although not to the same extent as fluorindole and chloroindole), but the rate of conversion to 5-bromotryptophan was very low. As with the 5-fluoroindole and 5-chloroindole reactions, 5-bromoindole was rapidly taken up by strains PHL644 and MC4100.

### Biofilm-mediated biotransformation

**Figure 5:**
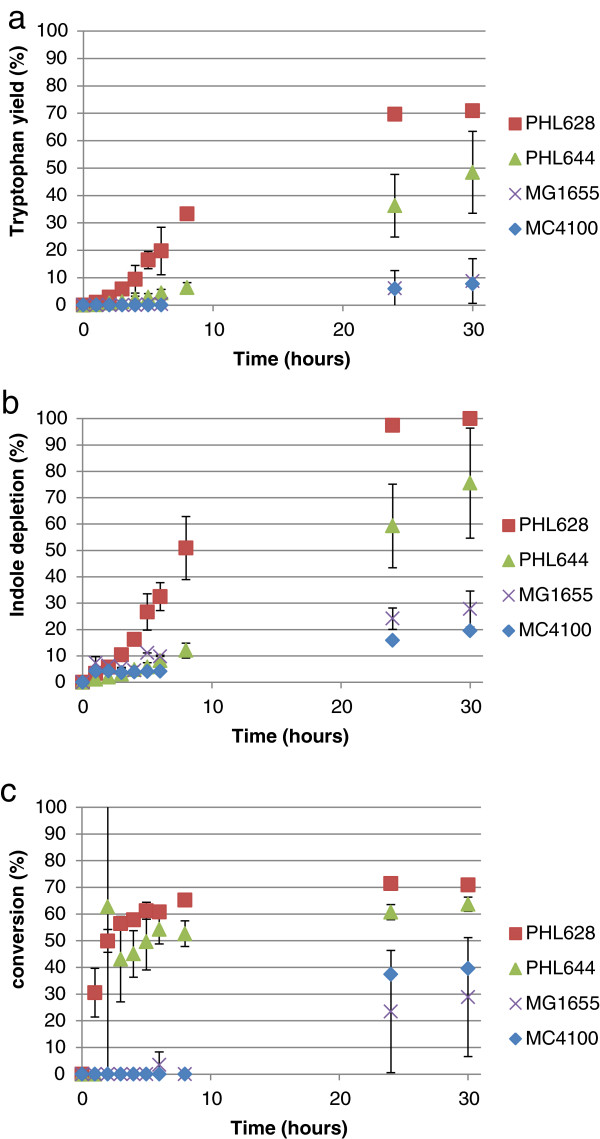
**Biotransformation of 5-fluoroindole to 5-fluorotryptophan using engineered biofilms comprising four strains.** Concentrations of 5-fluorotryptophan and 5-fluoroindole were measured using HPLC and percentage 5-fluorotryptophan accumulation **(a)**, percentage 5-fluoroindole depletion **(b)** and the selectivity of the 5-fluoroindole to 5-fluorotryptophan reaction **(c)** were plotted against time. All cells contained pSTB7.

Results for the biotransformation of 5-fluoroindole to 5-fluorotryptophan using engineered biofilms that had been matured for 7 days in M63 medium are shown in Figure 5. Biofilm-mediated reactions were dramatically different to planktonic reactions, both in terms of each strain’s relative activity but also in overall reaction kinetics. The rapid import of haloindole observed in planktonic MC4100 strains (Figures 3 and 4) was not observed in biofilm reactions, probably a consequence of the changes in indole transport and metabolism upon biofilm formation (Lee & Lee, 2010). Strains containing the *ompR234* mutation were all more catalytically active than their wild type counterparts; this is probably due in part to the lower entrapment of wild type cells (Figure 1). Unlike reactions performed with the cells in the planktonic state, the PHL628 pSTB7 biofilm outperformed PHL644 pSTB7 in terms of overall fluorotryptophan yield, rate of conversion and selectivity. MG1655 pSTB7 and MC4100 pSTB7 displayed minimal conversion of metabolised fluoroindole to fluorotryptophan until after 24 hours incubation (Figure 5c).

**Figure 6:**
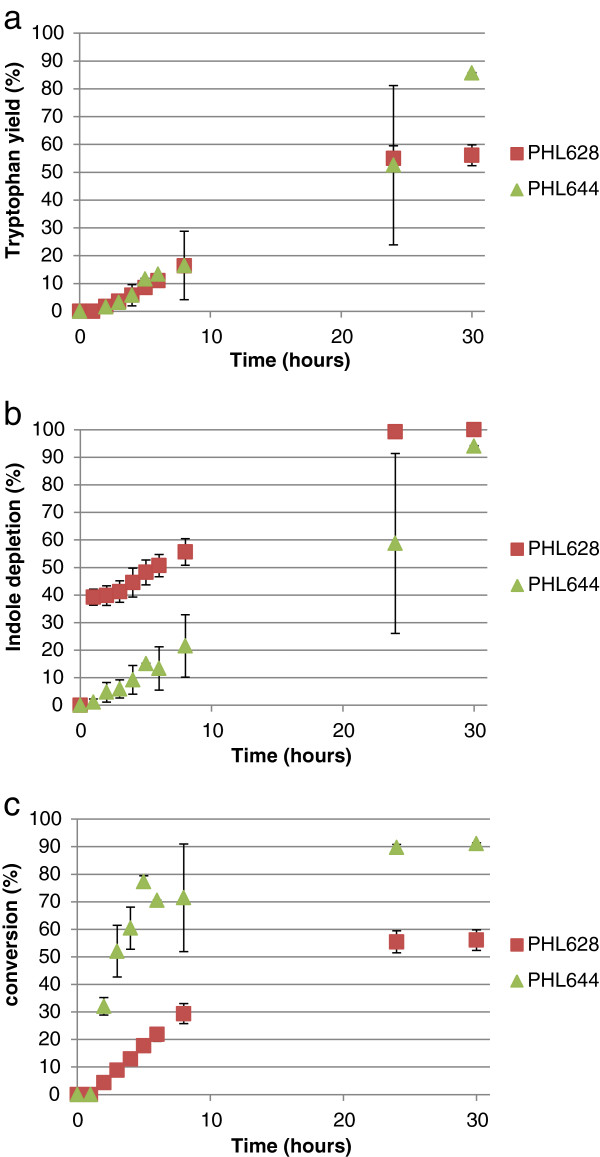
**Biotransformation of 5-chloroindole to 5-chlorotryptophan using engineered biofilms comprising two strains.** Concentrations of 5-chlorotryptophan and 5-chloroindole were measured using HPLC and percentage 5-chlorotryptophan accumulation **(a)**, percentage 5-chloroindole depletion **(b)** and the selectivity of the 5-chloroindole to 5-chlorotryptophan reaction **(c)** were plotted against time. All cells contained pSTB7.

For the biofilm-mediated conversion of 5-chloroindole to 5-chlorotryptophan (Figure 6), PHL628 pSTB7 displayed rapid 5-chloroindole import (similar to MC4100 planktonic cells). Conversion was higher in PHL644 pSTB7 than PHL628 pSTB7, probably a consequence of the earlier exhaustion of 5-chloroindole in the latter strain. As with the planktonic 5-bromotryptophan reactions, the yields of biofilm-catalysed 5-bromotryptophan biotransformations were very low; 5-bromoindole was taken up by cells, but converted to 5-bromotryptophan at a very low rate (Additional file 1: Figure S4).

In order to compare the biotransformation reaction on an equivalent basis between different strains and haloindoles, initial reaction rate data normalised by cell dry mass (expressed in units of μmol halotryptophan (mg dry cells)^-1^ h^-1^) are presented in Table 1. As previously observed (Tsoligkas *et al*., 2011), reaction rates followed the trend fluoroindole > chloroindole > bromoindole. Biofilms and planktonic cells had very similar initial reaction rates except for MG1655 pSTB7 and PHL628 pSTB7 for fluoroindole when the initial conversion rate using biofilms was three to four times that of planktonic cells. It should be noted that initial rates do not necessarily relate to overall reaction yields, and these data should be consulted in conjunction with Figures 3, 4, 5 and 6.

**Table 1:** **Summary of the initial rate of halotryptophan production expressed as μmol halotryptophan (mg dry cells)**^**-1**^ **h**^**-1**^

**Strain**	**5-fluoroindole**		**5-chloroindole**		**5-bromoindole**	
	**Planktonic**	**Biofilm**	**Planktonic**	**Biofilm**	**Planktonic**	**Biofilm**
MG1655 pSTB7	0.26	0.72	0.17	ND	0.13	ND
PHL628 pSTB7	0.28	1.08	0.19	0.16	0.08	0.05
MC4100 pSTB7	0.35	0.33	0.25	ND	0.05	ND
PHL644 pSTB7	0.73	0.65	0.43	0.37	0.06	0.07

*ND*, Not determined.

### Cell physiology during biotransformation reactions

To eliminate the possibility that differences in biotransformation yields were due to changes in bacterial viability or physiology, flow cytometry was used to determine the proportion of PHL644 pSTB7 cells with membrane potential and membrane integrity (i.e. live cells) after 2 and 24 hours of biotransformation reactions (Table 2). In all conditions, the vast majority of the cell population were live cells. Neither the presence of DMSO or any 5-haloindole had any detrimental effect on cell viability in planktonic biotransformations, even after 24 hours (p < 0.05). The presence of 5-haloindoles did not have a statistically significant effect on the percentage of biofilm cells alive after either 2 or 24 hours (p < 0.05); however, the proportion of live biofilm cells decreased between 2 and 24 hours (p < 0.05). Examples of plots obtained through flow cytometry are shown in Additional file 1: Figure S5.

**Table 2:** **Percentage (mean ± S.D.) of *E. coli *** **PHL644 pSTB7 cells that were alive determined using flow cytometry during biotransformations performed with planktonic cells or biofilms**

**Reaction conditions**	**Cell type and time of sampling**			
	**Planktonic**	**Planktonic**	**Biofilm**	**Biofilm**
	**2 hours**	**24 hours**	**2 hours**	**24 hours**
Reaction Buffer, 5% DMSO	99.52 ± 0.14	99.32 ± 0.40	95.73 ± 2.98	92.34 ± 0.10
Reaction Buffer, 5% DMSO, 2 mM 5-fluoroindole	99.38 ± 0.60	99.24 ± 0.80	96.44 ± 1.51	90.73 ± 0.35
Reaction Buffer, 5% DMSO, 2 mM 5-chloroindole	99.27 ± 0.33	99.33 ± 0.20	95.98 ± 2.64	91.69 ± 3.09
Reaction Buffer, 5% DMSO, 2 mM 5-bromoindole	99.50 ± 0.18	99.33 ± 0.20	96.15 ± 1.94	91.17 ± 2.19

## Discussion

### Biofilm formation

Biofilm formation is a complex process governed by many environmental cues, detected and coordinated through a complex regulatory network (Beloin *et al.*,^2008^). The osmolarity-sensing two component regulatory system EnvZ-OmpR is crucial to the regulation of biofilm formation in *E. coli* (Shala *et al.,*^2011^; Vidal *et al.,*^1998^). OmpR transcriptionally activates the *csgDEFG* operon; CsgD in turn activates transcription of the *csgBAC* operon, encoding the curli structural proteins which enable initial attachment of bacteria to surfaces (Prigent-Combaret *et al.,*^2001^; Ogasawara *et al.,*^2010^; Brombacher *et al.,*^2003^). In addition, CsgD also activates transcription of *adrA*, encoding a putative diguanylate cyclase which is predicted to generate c-di-GMP and thus activate cellulose production (Bhowmick *et al.,*^2011^). The *ompR234* mutation carried in strains PHL628 and PHL644 comprises a point mutation (L43R) located within the receiver domain, which enhances activation of *csgDEFG* (Prigent-Combaret *et al.,*^2001^; Prigent-Combaret *et al.,*^1999^; Vidal et al. 1998). It was, therefore, expected that the *ompR234* strains would form biofilm more readily than MC4100 and MG1655 (Figure 2).

Indole has previously been shown capable of enhancing biofilm formation (Chu *et al.,*^2012^; Pinero-Fernandez *et al.,*^2011^), whereas tryptophan has been shown to decrease biofilm formation (Shimazaki *et al.,*^2012^). Therefore the presence of pSTB7 could result in decreased biofilm formation since tryptophan concentrations (both intracellular and extracellular) could be predicted to be higher in cells containing pSTB7. *E. coli* MC4100 and MG1655 did not form substantial biofilms, hence the presence of pSTB7 did not have a significant effect on these strains (Figure 2). pSTB7 decreased the biomass of PHL628 biofilms, although it did not decrease biofilm formation in PHL644. This was possibly a consequence of the higher activity of tryptophan synthase in biofilms of PHL628 pSTB7 compared to PHL644 pSTB7 (Table 1), which would deplete intracellular indole.

### Biotransformation rates and efficiencies

As previously noted (Tsoligkas *et al.,*^2011^), the initial rate of biotransformation reactions followed the trend 5-fluorotryptophan > 5-chlorotryptophan > 5-bromotryptophan, irrespective of strain (Table 1); this has been ascribed to steric hindrance of the TrpBA enzyme by bulky halogen adducts (Goss and Newill, 2006). The selectivity of the haloindole to halotryptophan reaction was not 100% in any of the cases studied. In most cases, the reaction stopped due to haloindole depletion. Since, in the absence of pSTB7, haloindole concentrations did not decrease over the course of 30-hour biotransformation reactions, it can be concluded that all haloindole consumed by pSTB7 transformants was initially converted to halotryptophan by the recombinant TrpBA, and that haloindole influx into cells was driven by this conversion. Indole is thought to predominantly enter bacteria via diffusion through the membrane, a process which would probably be aided by the presence of DMSO in the reaction buffer (Pinero-Fernandez *et al.,*^2011^). Haloindole utilisation data (Figures 3b and 4b) reveal that MC4100 and its *ompR234* derivative PHL644 display an extremely rapid initial influx of haloindole within the first hour of planktonic reactions. This is not observed in planktonic reactions with MG1655 or PHL628, where indole influx is steadier. Initial halotryptophan production rates reflect these data (Table 1). Biofilm reactions display a different trend; rapid indole influx is only seen in PHL628 chloroindole reactions (Figure 6b), and indole influx is slower in PHL644 than PHL628. Again, this is probably due to the higher rate of halotryptophan production in biofilms of PHL628 than PHL644 (Table 1), driving haloindole influx via diffusion.

Since halotryptophan concentrations were measured here by HPLC in the cell-free extracellular buffer, all measured halotryptophan must have been released from the bacteria, either by active or passive processes. Therefore, conversion ratios of less than 100% must derive either from failure of halotryptophan to leave bacteria or alternative halotryptophan utilisation; the latter could be due to incorporation into proteins (Crowley *et al*., 2012) or degradation to haloindole, pyruvate and ammonia mediated by tryptophanase TnaA (Figure 1). Although regenerating haloindole, allowing the TrpBA-catalysed reaction to proceed again, this reaction would effectively deplete serine in the reaction buffer and so potentially limit total conversion. The concentration of serine could not be monitored and it was not possible to determine the influence of this reverse reaction. Deletion of *tnaA* would remove the reverse reaction, but since TnaA is required for biofilm production (Shimazaki *et al.,*^2012^) this would unfortunately also eliminate biofilm formation so is not a remedy in this system.

Synthesis of TnaA is induced by tryptophan, which could explain the decrease in conversion selectivity over time observed in planktonic MG1655 and PHL628 chlorotryptophan reactions (Figure 4c); chlorotryptophan synthesis could potentially induce TnaA production and thus increase the rate of the reverse reaction. In other reactions, selectivity gradually increased over time to a plateau, suggesting that initial rates of halotryptophan synthesis and export were slower than that of conversion back to haloindole.

Taken together, these observations are likely due to underlying differences between strains MG1655 and MC4100 and between planktonic and biofilm cells in terms of: indole and tryptophan metabolism, mediated by TrpBA and TnaA; cell wall permeability to indole; and transport of tryptophan, which is imported and exported from the cell by means of transport proteins whose expression is regulated by several environmental stimuli. They underline the requirement to assess biotransformation effectiveness, both in terms of substrate utilisation and product formation, in multiple strains, in order that the optimal strain might be selected.

We had previously hypothesised that biofilms were better catalysts than planktonic cells for this reaction due to their enhanced viability in these reaction conditions, allowing the reaction to proceed for longer; however, flow cytometry reveals this to be untrue. Therefore, the reasons for extended reaction times in biofilms as compared to planktonic cells must be more complicated. A second possible reason for such behaviour could the higher plasmid retention of biofilm cells (O’Connell *et al.,*^2007^) that could allow greater *trpBA* expression and thus more enzyme in biofilm cells. However, the initial rate of halotryptophan production per mass of dry cells were very similar in most of the cases apart from PHL628 pSTB7 and MG1655 pSTB7 for fluoroindole; therefore it appears that such hypothesis could be disregarded. Furthermore the similarity between the initial conversion rates between the two physiological states (biofilms and planktonic) suggests that mass transfer of haloindole through the biofilm was not the limiting step in the biotransformation because, if this was the case, lower initial conversion rates would have been found for biofilm reactions. Future studies will focus on the increased longevity of the reaction in biofilms when compared to planktonic cells, and the differences in tryptophan and indole metabolism in biofilms and planktonic cells.

In conclusion, in order to be used as engineered biofilms *E. coli* strains need to be able to readily generate biofilms, which can be achieved through the use of *ompR234* mutants. Despite the presence of native tryptophan synthase in *E. coli*, a plasmid carrying the *trpBA* genes under the control of a non tryptophan-repressed promoter was required to achieve detectable conversions of 5-haloindole to 5-halotryptophan. PHL644 pSTB7 returned the highest conversion when planktonic cells were employed in biotransformations but PHL628 pSTB7 gave the highest production of fluorotryptophan when biofilms were used. Higher viability is not the reason for biofilms’ greater performance than planktonic cells; complex differences in indole and tryptophan metabolism and halotryptophan transport in biofilm and planktonic cells probably determine reaction efficiency. The results underline that biotransformation reactions need to be optimised in terms of host strain choice, recombinant enzyme production and method of growth for the chosen biocatalyst.

## Competing interests

The authors declare that they have no competing interests.

## Supplementary Material

Additional file 1 Supplemental methods, Figures S1-S5 and Table S1. Click here for file
