# Supplementary material for: Optimisation of engineered Escherichia coli biofilms for enzymatic biosynthesis of l-halotryptophans
Source: AMB Express. 2013 Nov 4;3:66. doi: 10.1186/2191-0855-3-66 (PMC3843566; doi:10.1186/2191-0855-3-66)
Supplement: Additional file 1 — Supplemental methods, Figures S1-S5 and Table S1. [file 2191-0855-3-66-S1.docx]

# *AMB Express*

# Optimisation of engineered *Escherichia coli* biofilms for enzymatic biosynthesis of l-halotryptophans

Stefano Perni ^1^, Louise Hackett ^1^, Rebecca J.M. Goss ^2^, Mark J. Simmons ^1^,

Tim W. Overton ^1,^*

^1^ School of Chemical Engineering, University of Birmingham, Birmingham, B15 2TT, UK

^2^ School of Chemistry, University of St. Andrews, St Andrews, Fife, KY16 9ST, UK

*Corresponding author: [t.w.overton@bham.ac.uk](mailto:t.w.overton@bham.ac.uk)

Supporting information

Supplemental methods 2

Engineered biofilm preparation 2

Biotransformation conditions 2

Flow cytometry 3

HPLC 4

Supplementary figures (S1-S5) 5

Supplementary table S1 10

#### Supplemental methods

#### Engineered biofilms preparation and maturation

All *E. coli* strains in this study were investigated in the form of biofilms on glass microscope slides 48 mm x 25 mm obtained from a standard microscope slide (75 x 25 mm, VMR). Glass slides were washed with iso-propanol and dried for 30 min, then they were coated with 1 mL of 0.1% (w/v) PLL in water (Sigma) and left to dry in an oven at 60 °C overnight.

Following incubation, cultures were transferred aseptically into sterile 750 mL polypropylene centrifuge bottles (Beckman Coulter UK Ltd.) containing a bed of glass beads (200 g, soda-glass beads, 4 mm diameter) to provide a flat surface to prevent cracking during centrifugation, then the PLL-coated glass slides were placed on the glass beads and the cultures were centrifuged at 1851g for 10 minutes in a centrifuge (JOUAN 420) fitted with a swinging bucket rotor. After centrifugation, the glass slides were gently placed in 250 mL sterilised wide necked Erlenmyer flasks (Fisher Scientific) containing 100 mL of M63 medium (100 mM KH_2_PO_4_, 15 mM (NH_4_) _2_SO_4_, 0.8 mM MgSO_4_·7H_2_O, 9 mM FeSO_4_·2H_2_O, 1 mM glucose, adjusted to pH 7.0), supplemented with ampicillin (100 μg mL^-1^) for pSTB7 transformants. The spin coated biofilms were incubated in an orbital shaker incubator at 30 °C, 70 rpm with a throw of 19 mm for a maturation period of 7 days.

#### Biotransformation with Biofilm

Glass slides supporting *E. coli* biofilms were carefully removed from the M63 maturation medium. Planktonic cells were removed from the biofilm washing twice in 50 mL of sterile PBS, then the glass slides were placed in 250 mL sterilised wide necked Erlenmyer flasks (Fisher Scientific) containing 50 mL of reaction buffer (0.1 M KH_2_PO_4_, 7 mM Serine, 0.1 mM Pyridoxal 5′-phosphate (PLP), adjusted to pH 7.0, supplemented with 5% (v/v) DMSO) and haloindole as required. The flasks were placed in an orbital shaker incubator (30°C, 70 rpm with a 19 mm throw). 1 mL aliquots of the reaction buffer were taken every hour for the first 6 hours and then at regular intervals thereafter. Any reaction in the samples was stopped by centrifugation (16060g, 10 min) in order to remove any planktonic cells in solution. The concentration of 5-halotryptophan and 5-haloindole in each of the aliquots was determined by HPLC analysis.

#### Biotransformation with Planktonic Cells

*E. coli* cultures obtained after 24 hours growth at 30 °C in half LB medium were employed for planktonic cells reactions. 50 mL of suspension was transferred to sterile 50 mL tubes and centrifuged at 1851g for 10 minutes (JOUAN 420). The supernatant was removed and the pellets were resuspended in sterile phosphate buffer and centrifuged again. The liquid was removed and 25 mL of reaction buffer was added and the cells resuspended. The tubes were placed in an orbital shaker incubator (30°C, 70 rpm with a 19 mm throw). 1 mL aliquots of the reaction buffer were taken every hour for the first 6 hours and then at regular intervals thereafter. Any reaction in the samples was stopped by centrifugation (16060g, 10 min) in order to remove any planktonic cells in solution. The concentration of 5-halotryptophan and 5-haloindole in each of the aliquots was determined by HPLC analysis.

#### Determination of the initial rate of halotryptophan production

A linear regression was performed for the data of halotryptophan concentration during the first 3 hours of biotransformation and the slope of such line was considered the initial rate of halotryptophan production. This was divided by the biomass contained within each biotransformation to give the rate per unit biomass.

### Flow cytometry

A BD Accuri C6 flow cytometer was used. Cells were illuminated by a 488nm laser; forward and side scatter were measured along with green and red fluorescence using 533/30 and 670LP filters respectively. 10000 data points were collected for each sample at a data rate of 1000-2500 events s^-1^. Data were plotted on green versus red fluorescence plots (Figure S7) and gated according to cell physiology (PI^-^ BOX^-^ live cells; PI^-^ BOX^+^ intact cells without membrane potential; PI^+^ BOX^+^ dead cells). The analysis was performed on two independent samples for each condition studied and data are presented (Table 2) as mean ± standard deviation.

### Statistical analysis

Student’s t-test was employed to determine statistically significant differences between *E. coli* strain using MiniTab software and setting a p value of 0.05.

### HPLC

Timecourse showing increase of mobile phase (methanol) over time for HPLC analysis.

| Time (min) | % Methanol |
| --- | --- |
| 0-0.5 | 10 |
| 0.5-12.5 | 10-90 |
| 12.5-15 | 90 |
| 15-16 | 90-10 |
| 16-21 | 10 |


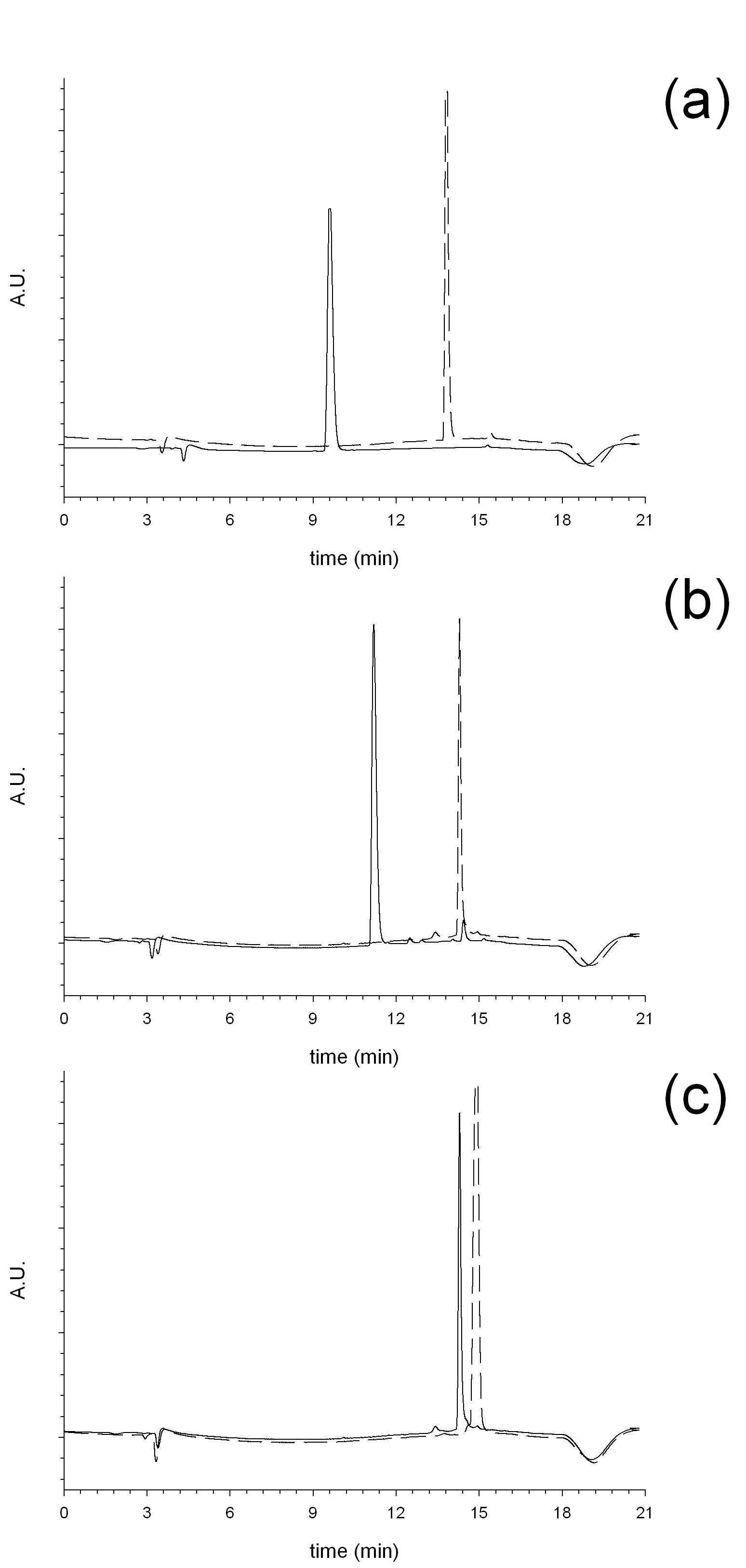


**Fig. S1** Examples of HPLC chromatograms for (a) Fluoro- (b) Chloro- and (c) Bromo-. Indole (dashed lines) and tryptophans (solid lines). X axes show retention times.


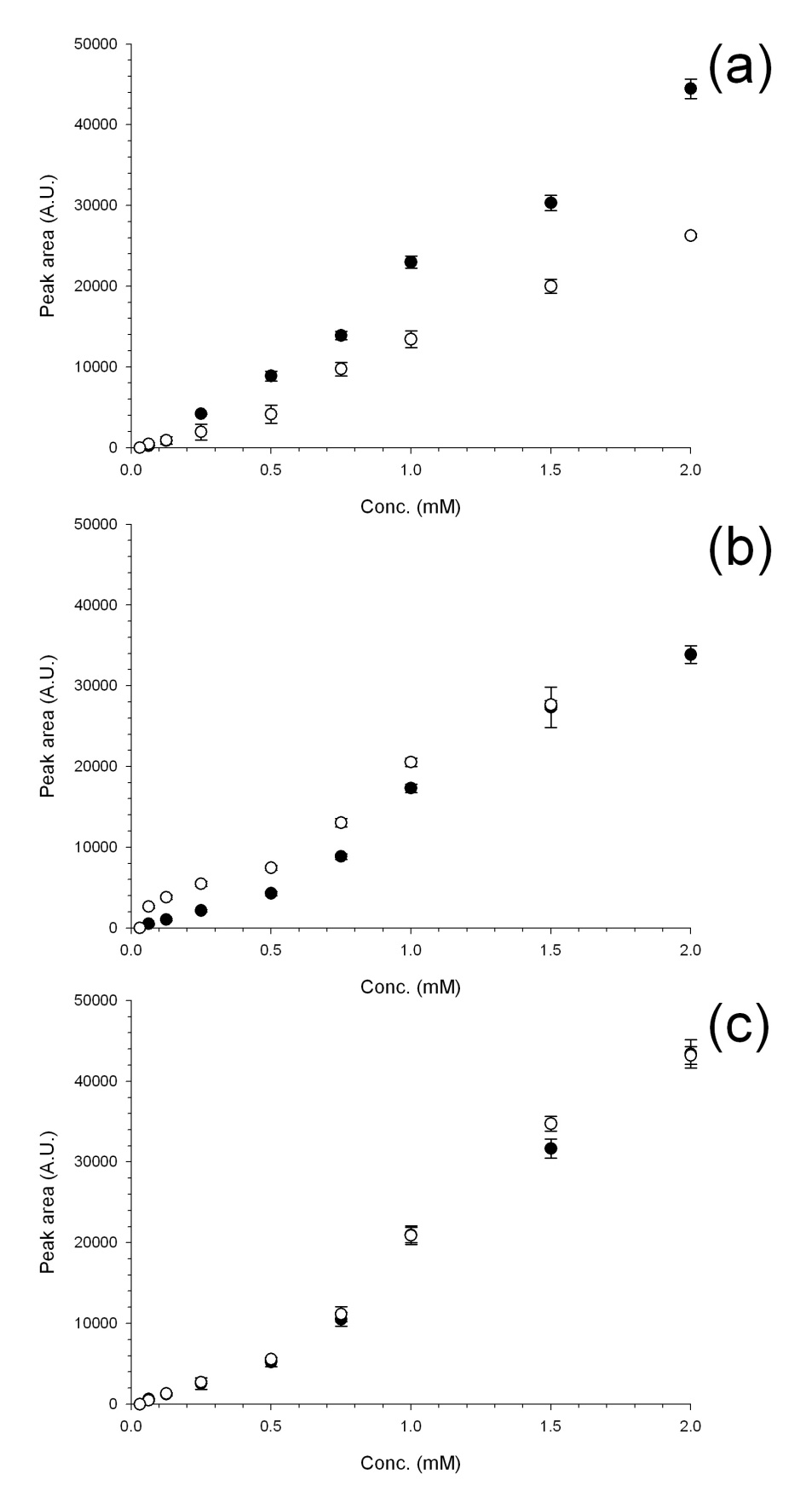


**Fig. S2** Relation between HPLC peak area and concentration of (a) (○) 5-fluoroindole and (●) 5-fluorotryptophan; (b) (○) 5-chloroindole and (●) 5-chlorotryptophan; and (c) (○) 5-bromoindole and (●) 5-bromotryptophan.

**Fig. S3** (a) percentage 5-bromotryptophan accumulation; (b) percentage 5-bromoindole depletion; and (c) selectivity of the 5-bromoindole to 5-bromotryptophan reaction for biotransformation catalysed by planktonic cells.

**Fig. S4** (a) percentage 5-bromotryptophan accumulation; (b) percentage 5-bromoindole depletion; and (c) selectivity of the 5-bromoindole to 5-bromotryptophan reaction for biotransformation catalysed by biofilms matured for 7 days.


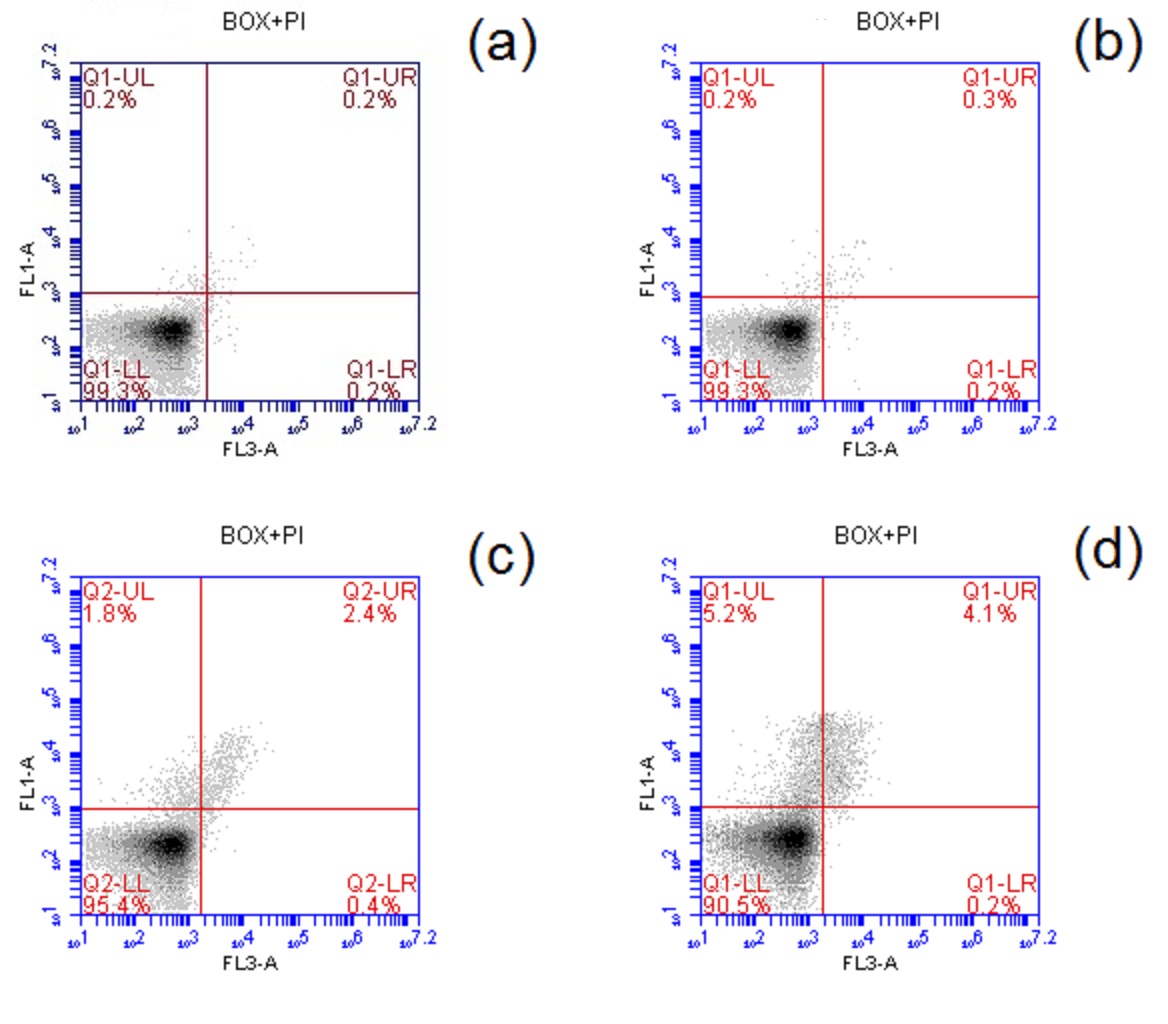


**Fig. S5** Examples of flow cytometry plots during biotransformation for planktonic cells after (a) 2 hours and (b) 24 hours or biofilm cells after (c) 2 hours and (d) 24 hours. The x axis shows red fluorescence from propidium iodide, the y axis green fluorescence from bis-oxanol. Cells in each quadrant have the following properties: lower-left, membrane potential and integrity, therefore alive; upper-left, membrane integrity but no membrane potential, therefore injured; upper-right, neither membrane potential nor integrity, therefore dead.

|  | Planktonic reactions | | | Biofilm reactions | | |
| --- | --- | --- | --- | --- | --- | --- |
| Strain | Fluoroindole | Chloroindole | Bromoindole | Fluoroindole | Chloroindole | Bromoindole |
| PHL628 | 2.26 | 0.66 | 0.37 | 2.56 | 1.58 | 0.73 |
| PHL644 | 2.48 | 0.78 | 0.44 | 2.26 | 0.47 | 0.34 |
| MC4100 | 2.48 | 0.78 | 0.44 | 1.50 | ND | ND |
| MG1655 | 2.26 | 0.66 | 0.37 | 1.50 | ND | ND |

**Supplementary Table S1.** Initial concentrations (in mM) of 5-haloindole in each biotransformation reaction. Initial concentration was dependent upon the solubility of each indole. ND: not determined.
